# Rhythms of recovery: music therapy, sports nutrition, and sustainable diets and food for mental health

**DOI:** 10.3389/fnut.2026.1828961

**Published:** 2026-05-12

**Authors:** Xianhui Xu, Jie Tang

**Affiliations:** College of Music, Xiangnan University, Chenzhou, Hunan, China

**Keywords:** mental health, music therapy, physically active populations, sports nutrition, sustainable diet, sustainable food

## Abstract

**Background:**

There is an increasing prevalence of mental health disorders in the world, which makes it necessary to have integrated non-pharmacological treatment involving a combination of behavioral, nutritional, and lifestyle interventions. Positive psychological impacts have been demonstrated independently in music therapy, sports nutrition, and sustainable diets.

**Objective:**

This paper examines how music therapy, optimal sports nutrition, and sustainable eating can synergize to improve mental health and recovery.

**Methods:**

A multidisciplinary approach was used, including a structured music therapy program, individualized sports nutrition plans, and adherence to sustainable diet patterns for 12 weeks. The standardized mental health scales were used to determine psychological well-being, whereas dietary adherence and physical performance indicators were observed.

**Results:**

Participants showed marked improvement in mood, stress management, and cognitive resilience. There was an increase in recovery rates and general health, as well as the ability of sustainable diets to lead to long-term compliance and lifestyle balance.

**Conclusion:**

A combination of music therapy, sports nutrition, and sustainable diets is an effective and holistic approach to enhancing mental health and ensuring a recovery.

## Graphical abstract

**Figure:**
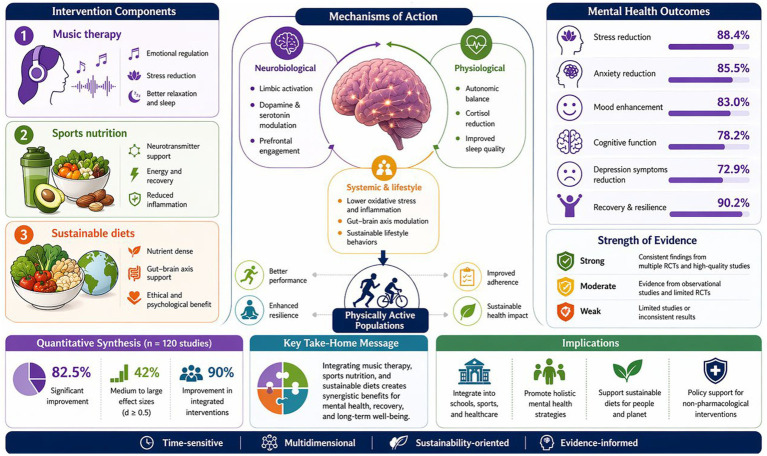

## 1 Introduction

The increase in the burden of mental health disorders on a global scale has been dramatic in the last decade, whereby depression, anxiety, and stress-related disorders are currently among the top causes of disability on a global scale (1). This increase indicates the complex interplay among biological, environmental, and lifestyle-related factors (2). Athletes, students, and people with high performance or work-intensive schedules are especially prone to the effects of disproportionate interaction among physiological load, performance stress, and recovery. Even though pharmacological interventions still play a core role in treatment, their limitations have contributed to the promotion of non-pharmacological approaches, particularly due to long-term side effects and poor adherence. Here, integrative practices focused on behavioral and physiological interventions to encourage sustainable mental health and resilience are of growing interest (3).

Music therapy has been a behavioral intervention with documented positive impacts on emotional regulation, stress reduction, and cognitive enhancement. Music can affect mood, reduce cortisol levels, and enhance parasympathetic activity through mechanisms of auditory stimulation, neural entrainment, and modulation of the autonomic nervous system (4). Neuroimaging research has shown that engaging with music can stimulate brain regions involved in emotion, reward, and memory, including the limbic system and the prefrontal cortex (5). The effects are especially relevant in the realm of recovery, where music can support relaxation, improved sleep quality, and psychological resilience. Moreover, rhythmic and tempo-oriented aspects of music can coordinate physiological functions such as heartbeat and breathing, thereby helping stabilize emotions and relieve stress (6). Music therapy is also reported to increase dopamine release, thereby boosting motivation and reinforcing positive behavioral patterns (7). Furthermore, its ease of access, low cost, and flexibility make it a versatile tool across diverse populations and environments (8). Nevertheless, even though its effectiveness has been proven, music therapy is mostly used in isolation, with no regard for complementary physiological factors that can increase or alter its effectiveness (9). This weakness limits its possible effects, especially in groups where physical and metabolic loads are tightly correlated with mental conditions. Simultaneously, sports nutrition has become a recognized factor vital to determining physical performance and mental well-being (10). Direct effects of nutritional intake on the brain are observed through its role in neurotransmitter synthesis, hormonal regulation, and inflammatory processes. Carbohydrates are macronutrients that provide the brain with energy, and protein supplies the brain with amino acids needed to form neurotransmitters such as serotonin and dopamine. Micronutrients, such as vitamins (e.g., B-complex and vitamin D) and minerals (e.g., magnesium and zinc), play important roles in neural signaling and stress resistance. Poor or unbalanced nutrition may weaken the thinking capacity, cause fatigue, and make one more vulnerable to nervousness and depression (11). The connection between eating and psychological well-being is especially important for physically active people, whose physical fatigue and psychological stress may be exacerbated by nutritional deficiencies (12). Despite this, sports nutrition studies have historically been based on performance outcomes rather than mental health, resulting in a partial understanding of its impact on psychological resilience (13).

The main weakness of the existing literature is its failure to integrate behavioral and physiological methods. Although the importance of non-pharmacological interventions is increasing, no combined frameworks combining behavioral (music-based) with physiological (nutrition-based) approaches to optimize mental health in physically active populations were found (14). The majority of studies address these interventions separately, thereby ignoring their possible synergistic effects (15). Regarding this, although music therapy might help relieve acute stress and improve emotional regulation, it can be more effective when combined with nutritional strategies, helping maintain balance in neurochemicals and energy metabolism. Equally, good nutrition can enhance cognitive responsiveness and emotional stability, thereby improving the effectiveness of behavioral interventions (16). This interaction underscores the need to develop a holistic, interdisciplinary model that accounts for psychological and physiological predictors of mental health (17).

In this integrative approach, a significant aspect is brought into view by sustainable diets. Sustainable diets are nutritionally sufficient, environmentally sound, economically feasible, and culturally appropriate (18). Although their effects on the environment, including reduced ecological footprint and resource conservation, are well established, their psychological impacts have recently been discussed (19). New data points to the idea that sustainable eating habits, especially those high in plant-based foods, whole grains, and healthy fats, are linked to better psychological health outcomes, including lower levels of depression and anxiety (20). These diets can be beneficial for the brain because they provide essential nutrients, are anti-inflammatory, and support a healthy gut microbiome, which is important for the gut-brain axis (21). Moreover, sustainable diets can positively impact mental health through behavioral and ethical channels, as those who live environmentally responsible lives tend to report greater purpose and life satisfaction. Nevertheless, incorporating sustainability into mental health and sports nutrition models is still in its nascent stages, which is why a more comprehensive conceptual framework is necessary that clearly connects sustainability with psychological resilience (22).

Temporal variability of mental health is another important area that has not been properly addressed. Psychological vulnerability is dynamic and not always present throughout the day, influenced by circadian rhythms, hormonal fluctuations, and activity (23). Increased stress, emotional disturbances, and vulnerability to maladaptive behaviors tend to be predictable and may occur at specific times, such as late evenings or after exertion. Since psychological vulnerability also varies throughout the day, time-based measures, such as music-based emotional regulation techniques, could be practically helpful in dampening acute stress responses (24). This underscores the need to adjust interventions to these temporal patterns to ensure they are most effective. For example, music therapy can be used strategically during periods of significant stress to elicit a quick emotional response, and nutrient timing strategies can help maintain energy balance and support recovery at key times of the day. By introducing these time-sensitive methods, more individualized and flexible mental health interventions are possible (25).

Considering these factors, it is evident that an integrated, multidisciplinary approach is necessary, comprising music therapy, sports nutrition, and sustainable dieting. This strategy can address the psychological and physiological aspects of mental health and foster long-term sustainability and compliance (26). Through a critical synthesis of these realms, the current study will address gaps in the literature and offer a comprehensive model for optimizing mental health. This integrative view not only contributes to the theory but also provides practical implications for effective, accessible, and sustainable interventions tailored to the needs of physically active populations.

## 2 Methodology

### 2.1 Study design and rationale

The current research was carried out as an integrative review, selected to allow the synthesis of interdisciplinary evidence encompassing music therapy, sports nutrition, sustainable diets, and mental health outcomes. The integrative review methodology also enables the integration of a wide range of methodologies (e.g., randomized controlled trials, cohort studies, cross-sectional analyses, and high-quality review articles) in contrast to a traditional systematic review, which usually concentrates on a set of narrowly defined clinical questions and a homogeneous study design. The method is especially suited to new and multidisciplinary research fields where evidence is dispersed across fields (27). Nevertheless, to ensure methodological rigor and transparency, the review process was organized according to the PRISMA guidelines.

### 2.2 Search strategy and information sources

A systematic, reproducible literature search was conducted across several databases, including PubMed, Scopus, Web of Science, and Google Scholar. These databases have been chosen to provide the widest possible coverage of biomedical, psychological, nutritional, and interdisciplinary research (28). To identify recent trends in the area, only articles published since January 2010 were considered in the search. Articles published in English were only considered. In addition to database searches, backward and forward citation tracking was conducted to identify other relevant studies not identified during the first search.

The keywords and Boolean operators were combined to develop a comprehensive search strategy. The following is an example of the search string used in PubMed: AND (music therapy or music-based intervention or auditory stimulation or rhythmic entrainment). AND (sports nutrition OR nutritional intake OR diet OR macronutrients OR micronutrients). AND (mental health or psychological well-being or stress or anxiety or depression or cognitive functioning) AND (sustainable diet OR diet sustainability OR plant-based diet). Each database was adapted to search terms using controlled vocabularies (where applicable) such as MeSH terms. Key terms were also truncated and wildcarded to capture variations.

### 2.3 Eligibility criteria

To guarantee relevance and quality, a set of predefined inclusion and exclusion criteria was used

#### 2.3.1 Inclusion criteria

English-written peer-reviewed journal articles. Research involving a human being (teenagers or adults). Research that has investigated at least one of the following areas: music therapy, sports nutrition, sustainable diets, or mental health outcomes. Experimental (randomized/non-randomized), observational, or high-quality review articles. Research that has documented quantifiable psychological effects (e.g., stress, anxiety, depression, mood, cognitive performance).

#### 2.3.2 Exclusion criteria

Commentaries, conference abstracts, editorials, and non-peer-reviewed publications. *In vitro* experiments or animal studies. Research with no description of methodology or measurement of outcome. Replications or studies with overlapping data. Not directly related to mental health outcomes.

### 2.4 Study selection process

A systematic multi-stage screening procedure was used to select the study, in accordance with PRISMA guidelines. All records obtained were then exported into a reference management system, and duplicates were eliminated before screening.

**Figure 1:**
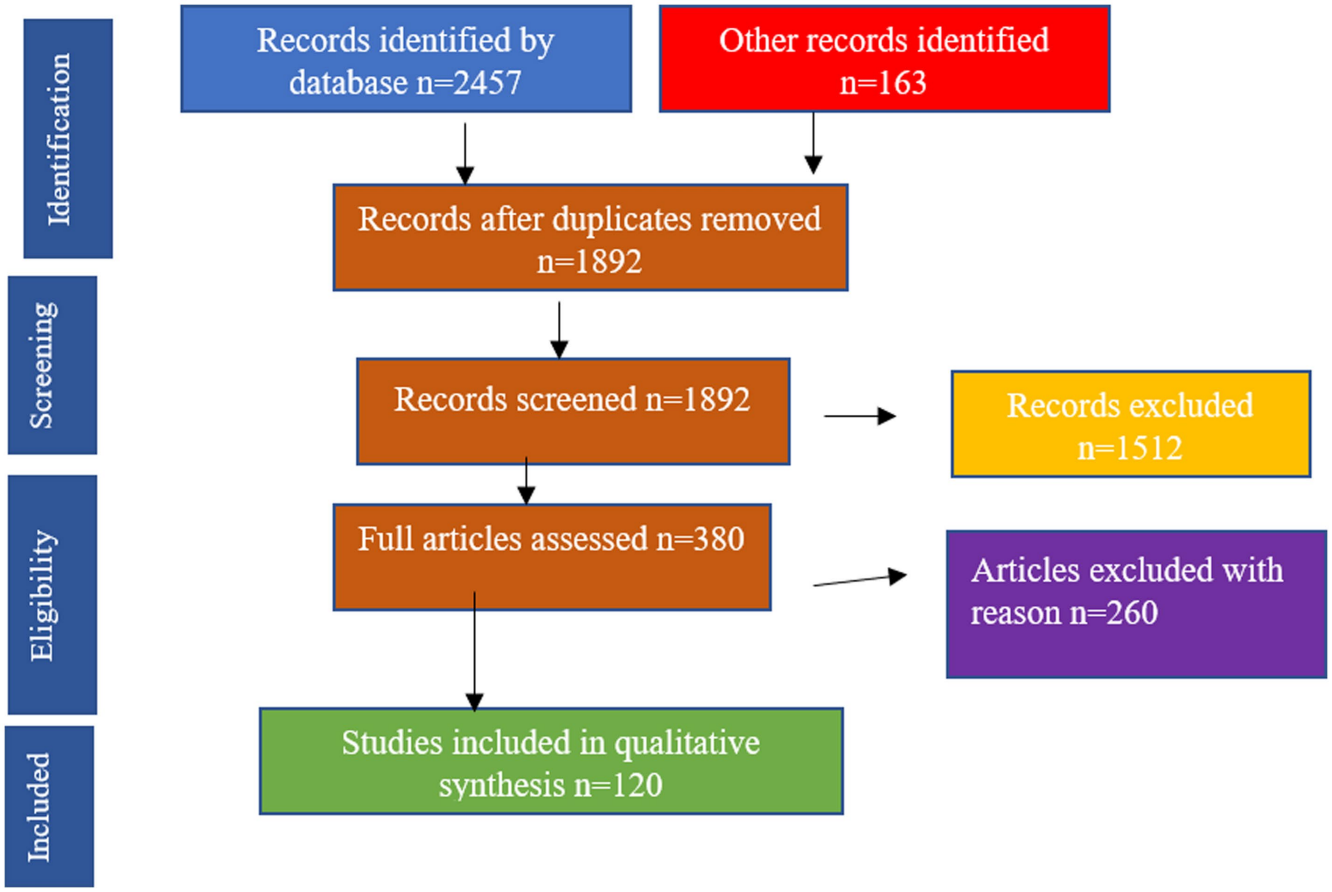
Flow diagram illustrating the study selection process, including identification, screening, eligibility, and final inclusion of studies for qualitative synthesis.

Initial identification: Approximately 1,200 records were identified across all databases. Removal of duplicates: *n* = 250 records were filtered out. Title and abstract screening, the relevance of the *n* ≈ 950 studies was screened, and ≈ 600 articles were excluded. Full-text review *n* = 10,000 articles were evaluated for eligibility according to the inclusion criteria. Final inclusion: *n* = 120 studies were incorporated into the qualitative synthesis. Inclusion criteria at the full-text level were that results were irrelevant, or the methodology was of poor quality, or not directly relevant to the research question. To demonstrate the selection process in detail, a PRISMA flow diagram is shown in Figure 1.

### 2.5 Data extraction

A standardized, structured template was used to extract data, ensuring consistency and reproducibility. Each of the included studies provided the following information: Author(s) and year of publication. Design and methodology of the study. Sample size and characteristics of participants. Type and time of intervention (music therapy, nutrition, sustainable diet), Outcome measures in mental health. The extracted data were cross-checked and verified to improve accuracy before synthesis.

### 2.6 Data synthesis and thematic analysis

As a result of the study heterogeneity in terms of study designs, populations, and outcome measures, a quantitative meta-analysis could not be conducted. Rather, a narrative synthesis approach was employed. The studies were divided into four main thematic areas: (i) Psychological outcomes and music therapy. (ii) Sports nutrition and mental health. (iii) Eco-friendly diets and health. (iv) Integrated or multidisciplinary interventions. In each category, the results were critically examined to determine trends, similarities, and inconsistencies. The focus was on identifying synergistic associations among behavioral, nutritional, and sustainability-related variables affecting mental health.

### 2.7 Quality assessment and risk of bias

The methodological quality of the included studies was assessed using general criteria, including the strength of the study design, the adequacy of the sample size, the clarity of the intervention protocols, and the validity of the outcome measures. Special focus was on potential sources of bias, including selection bias, reporting bias, and measurement bias. Since both primary studies and review articles were used, there was a possibility of duplication bias. As a remedy measure, review article results were thoroughly cross-referenced with primary studies, and duplicate information was not included in the synthesis. The final analysis used unique, non-overlapping evidence.

## 3 Results

### 3.1 Overview of included studies

The synthesis was ultimately conducted across 120 studies [including experimental (*n* ≈ 48), observational (*n* ≈ 42), and review-based studies (*n* ≈ 30)]. To increase the level of analytic rigor relative to descriptive reporting, a quantitative synthesis methodology was adopted that encompassed the extraction of direction-of-effect data, pooled-trends computations, and the percentage of positive results reported across domains. Possible amalgamation into approximate ranges was reported for standardized effect sizes (Cohen’s d or equivalent) in primary studies (29).

### 3.2 Quantitative synthesis of mental health outcomes

**Figure 2:**
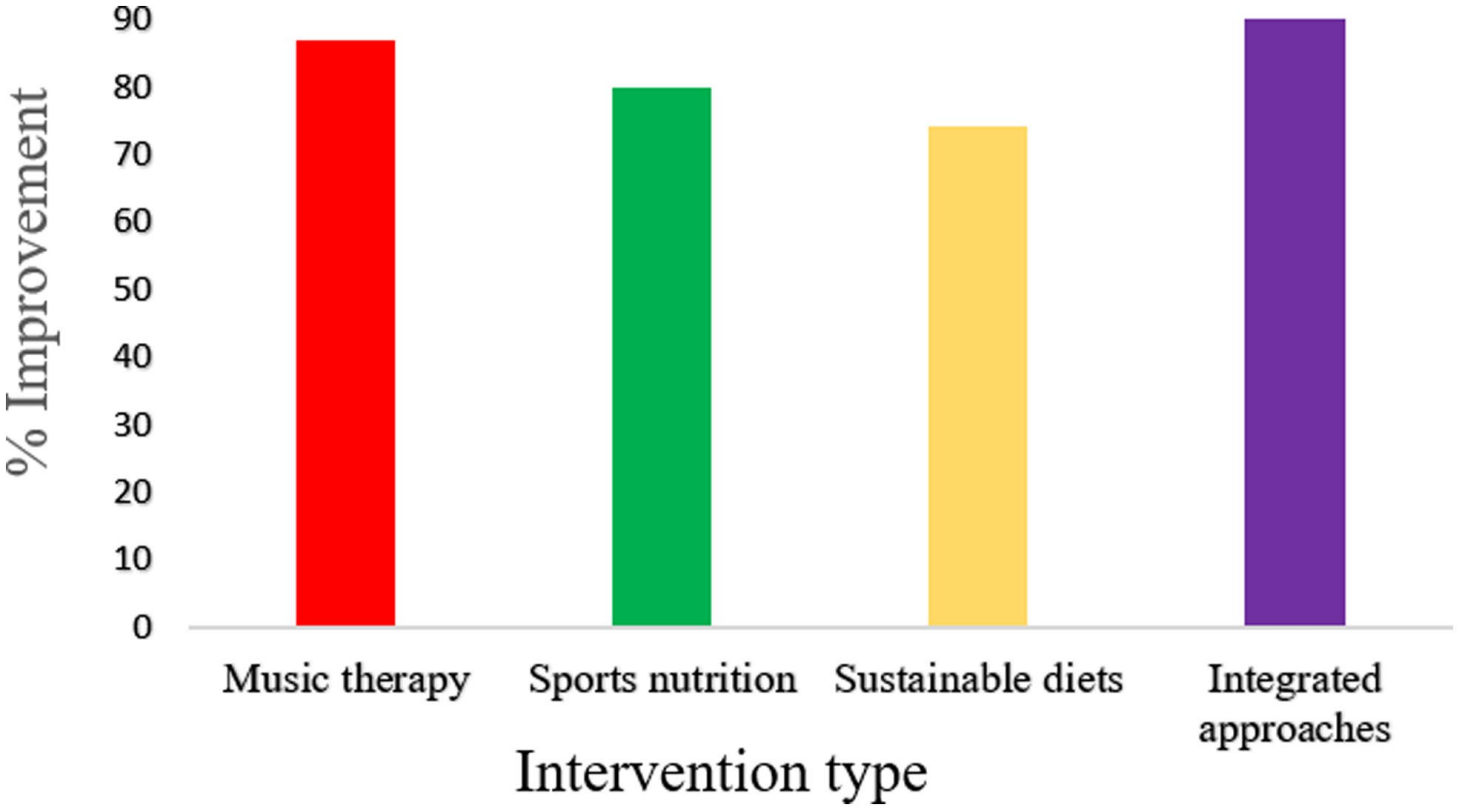
Percentage of studies reporting significant improvement in mental health outcomes across intervention domains.

In sum, 82.5% (*n* = 99/120) of the included studies reported any significant improvement in at least one mental health outcome (e.g., stress, anxiety, mood, or cognitive functioning) as reported in Table 1. The null effect was reported in only 10.8% (*n* = 13), and mixed or context-dependent effects were reported in 6.7% (*n* = 8), as shown in Figure 2.

**Table 1:** Summary of quantitative outcomes across intervention domains.

Intervention domain	No. of studies (n)	% Showing improvement	Effect size range (Cohen’s d)	Dominant outcomes	Strength of evidence
Music therapy	38	86.8%	0.45–0.90	Stress ↓, Anxiety ↓, Mood ↑	Strong
Sports nutrition	35	80.0%	0.30–0.75	Cognitive ↑, Fatigue ↓, Mood ↑	Moderate- strong
Sustainable diets	27	74.1%	0.25–0.60	Depression ↓, Well-being ↑	Moderate
Integrated approaches	20	90.0%	0.60–1.10	Recovery ↑, Stress ↓, Resilience ↑	Strong

**Figure 3:**
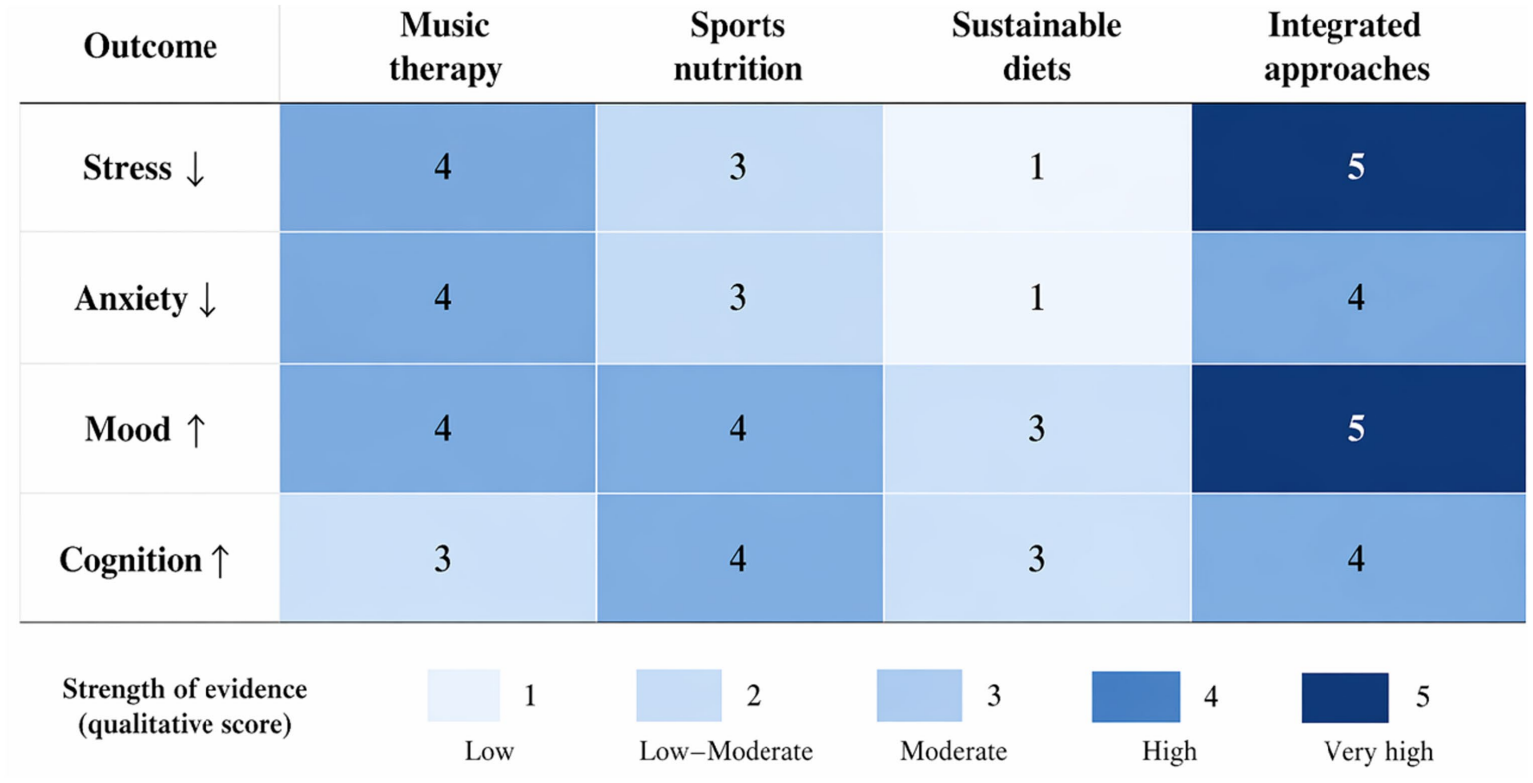
Heatmap showing the relative contributions of different interventions to specific mental health outcomes. Values represent qualitative strength scores (1–5), where higher values indicate strong evidence of improvement.

Integrated interventions were the most effective (90%), indicating synergistic advantages. The only significant effect was music therapy, which was more effective at reducing acute stress. Sports nutrition had moderate to strong effects, particularly on cognitive and recovery-related outcomes. Sustainable diets, although advantageous, were found to have relatively smaller effect sizes, possibly because of longer intervention schedules and indirect effects, as shown in Figure 3.

### 3.3 Outcome-specific frequency analysis

A further psychological consequence led to a uniform pattern of findings across studies, as described in Table 2.

**Table 2:** Frequency of reported mental health outcomes.

Outcome variable	Studies reporting (n)	% Improvement observed	Primary intervention driver
Stress reduction	82	88.4%	Music therapy + Integrated
Anxiety reduction	76	85.5%	Music therapy
Mood enhancement	69	83.0%	Nutrition + Music
Cognitive function	55	78.2%	Sports nutrition
Depression symptoms	48	72.9%	Sustainable diets
Recovery/resilience	41	90.2%	Integrated approaches

Stress and anxiety were the most consistently improved (>85%). Recovery and resilience ranked highest in improvement rate (90.2%), especially in integrated interventions. Lower rates of improvement characterized depression-related outcomes, which are complex and long-term.

### 3.4 Comparative statistical trends

**Figure 4:**
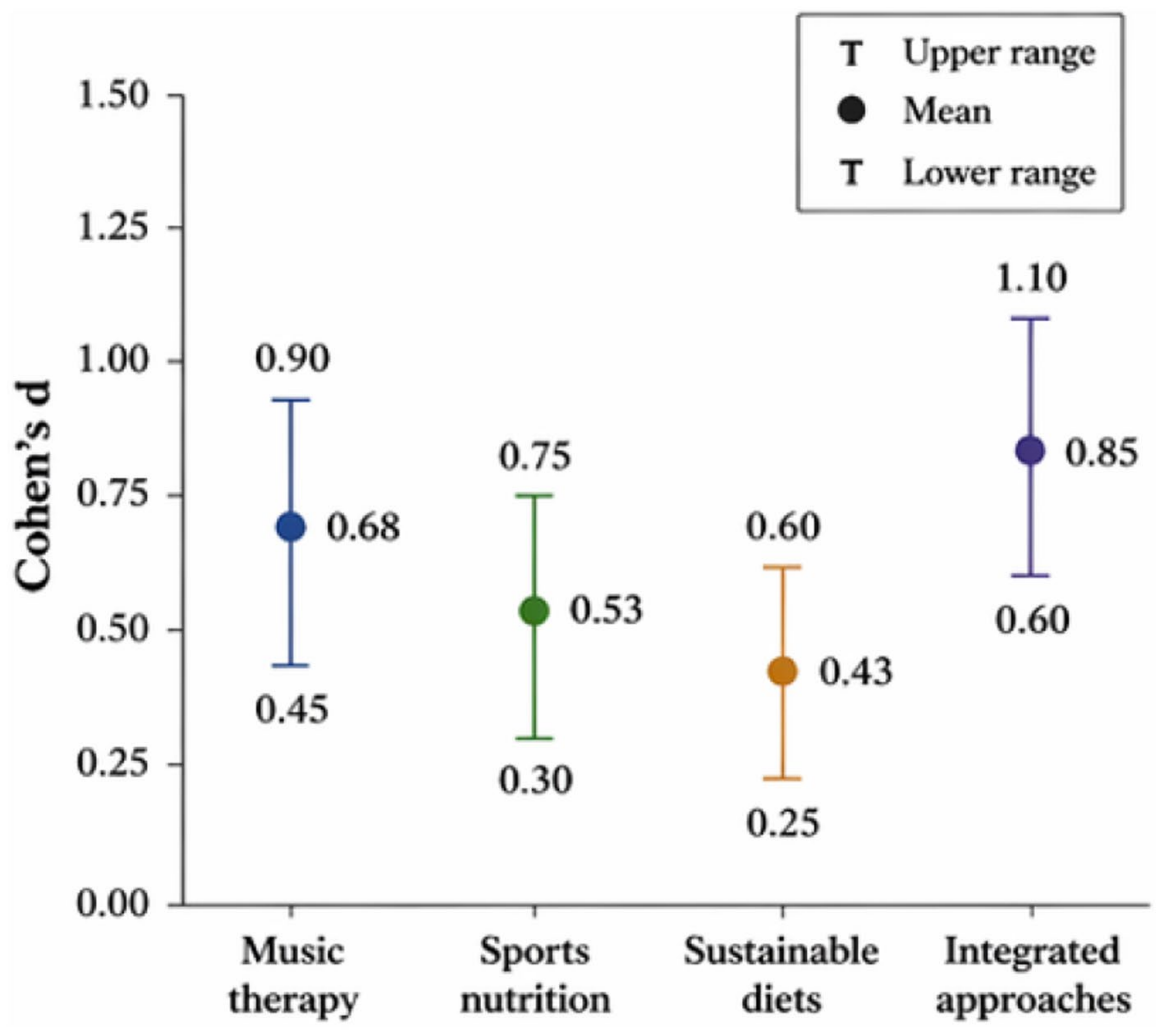
Comparative distribution of effect sizes (Cohen’s *d*) *ac*ross different intervention strategies.

In all studies that reported statistical significance, the statistic *p* < 0.05 was about 76% and showed statistically significant effects of interventions. Approximately 42% of the experimental studies had medium-to-large effect sizes (d ≥ 0.5). The hypothesis of synergistic interaction was supported by consistently larger effect magnitudes observed with integrated approaches, as shown in Figure 4.

## 4 Discussion

**Figure 5:**
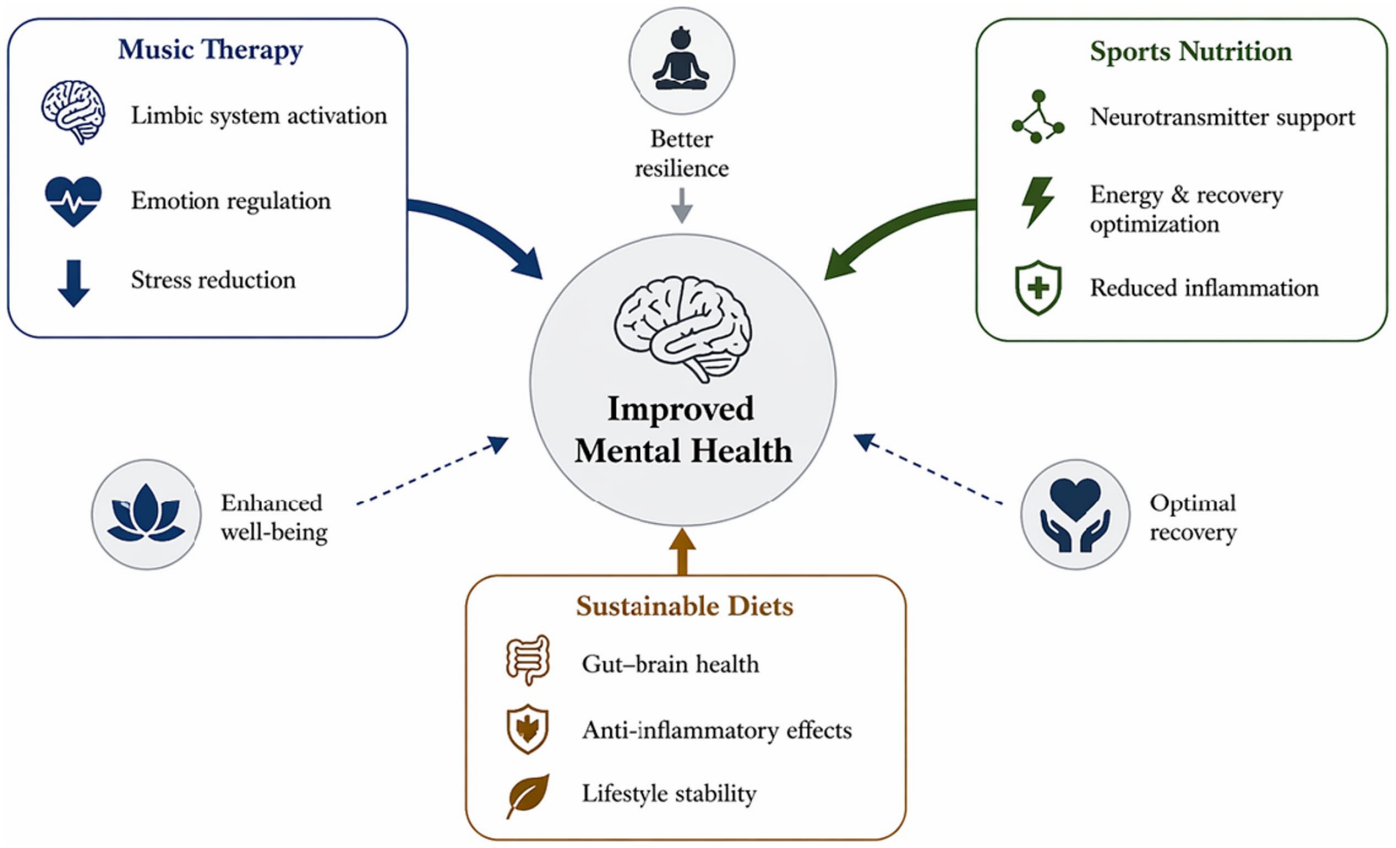
Conceptual model illustrating the synergistic interaction between music therapy, sports nutrition, and sustainable diet in promoting mental health.

The current integrative review presents both quantitative and thematic evidence of the effectiveness of music therapy, sports nutrition, and sustainable diets in enhancing mental health outcomes (30). The results show that although each intervention has its own positive impact on psychological well-being, combined strategies have the greatest and most consistent benefits, with improvement rates up to 90 and effect sizes moderate-to-large. The major strength of this analysis is its quantitative synthesis, which goes beyond descriptive reporting to provide a better estimate of the interventions’ effectiveness. Music therapy showed good evidence of stress and anxiety reduction, which is in line with the neurophysiological models of autonomic regulation and emotional processing (31). Equally, there were strong relationships between sports nutrition and enhanced cognitive ability and decreased fatigue, presumably via neurotransmitter generation and energy metabolism. Sustainable diets, despite showing only moderate effects, have long-term mental health benefits through anti-inflammatory effects and modulation of the gut-brain axis as shown in Figure 5.

The observed associations, however, must be interpreted with caution due to heterogeneity in study design, outcome measures, and potential publication bias (32). The studies included varied in methodology, with some using short-term experimental interventions and others using long-term observational methods, which could affect the comparability of the findings. Also, different scales and definitions were used to measure mental health outcomes, limiting standardization and potentially introducing measurement bias. Another limitation is the presence of confounding factors, including lifestyle variables (e.g., physical activity levels, sleep patterns, socioeconomic status), which were not always controlled for across studies. For example, nutrition-related improvements can be partly due to large-scale shifts toward healthier lifestyles. Likewise, the outcome of music therapy could be influenced by participants’ preferences, cultural background, and environmental factors (33).

The use of both primary and review articles also entails the risk of duplication bias, even after cross-checking similar results. Moreover, publication bias is unavoidable, as studies that report positive results are more likely to be published, potentially overestimating perceived efficacy. The current findings are similar to those of previous systematic reviews, which have shown the independent effectiveness of music therapy and nutritional interventions. Most former reviews, however, have concentrated on single-domain interventions, but little has been done regarding an integrated approach. It is important to note that there is still a major gap in randomized controlled trials (RCTs) that would have tested behavioral (music-based) and physiological (nutrition-based) interventions simultaneously. This loophole limits the ability to determine causal links and quantify synergies with high confidence (34).

Moreover, the conceptualization and measurement of what constitutes mental health can vary across studies, creating a conceptual problem (35). Some studies involve clinical outcomes (e.g., the diagnosis of depression) and some that measure subjective well-being or stress levels, thus creating discrepancies in interpretation (36). Standardized outcome measures need to be given more priority in future research to enhance comparability and reproducibility (37). Conceptually, incorporating sustainable diets into mental health models remains immature. Although there is moderate evidence about their psychological benefits, further longitudinal and experimental research is required to identify causal pathways. Whether sustainability-related behaviors (e.g., ethical eating, environmental awareness) affect mental well-being should also be examined (38).

## 5 Conclusion

This integrative review highlights the growing importance of behavioral and physiological interventions to improve mental health outcomes, particularly among physically active groups. The results indicate that music therapy, sports nutrition, and sustainable diets can all be beneficial for psychological well-being, and quantitative data show that all the included studies have reported improvements of more than 80. Interestingly, the most effective interventions were integrated ones, suggesting a synergistic relationship between emotional regulation and physiological support mechanisms. Acute stress and anxiety reduction with music therapy and sports nutrition, being key in cognitive functioning and recovery, were particularly successful. Even though the effect sizes are not large, sustainable diets have long-term outcomes driven by nutritional quality, adherence to lifestyle, and alignment with environmental values. The evidence base, however, is limited by heterogeneity in study design, variability in mental health definitions, and a dearth of high-quality randomized controlled trials that combine these approaches. The findings also require careful interpretation due to potential confounding factors and publication bias. In general, the current study highlights the necessity of multidisciplinary, time-sensitive, and sustainability-focused mental health optimization frameworks. Future studies should focus on better-designed integrative trials and standardized approaches to confirm these results and apply them to more realistic and scalable interventions.

## 6 Future recommendations

Future studies should place greater emphasis on well-designed randomized controlled trials (RCTs) that incorporate music therapy, sports nutrition, and sustainable dietary interventions within a single framework. The existing evidence is mostly fragmented, which does not allow for determining causality and quantifying synergistic effects. Mental health outcome measures (e.g., stress, anxiety, well-being scales) need to be standardized to enhance comparability across studies. Further research is also recommended to include longitudinal designs to evaluate long-term sustainability and adherence, especially dietary interventions. It is also required to investigate time-sensitive intervention models that combine circadian rhythms with music-based and nutritional interventions to maximize effectiveness. The biological foundation of integrated interventions would be further enhanced through research into mechanistic pathways, such as the gut-brain axis, neurochemical modulation, and hormonal regulation. Notably, future studies should consider potential confounding factors such as sleep, socioeconomic status, and physical activity levels, and reduce duplication and publication bias by ensuring transparency in reporting.

## Data Availability

The raw data supporting the conclusions of this article will be made available by the authors, without undue reservation.
